# A general strategy to determine the congruence between a hierarchical and a non-hierarchical classification

**DOI:** 10.1186/1471-2105-8-442

**Published:** 2007-11-15

**Authors:** Antonio Marco, Ignacio Marín

**Affiliations:** 1 Departamento de Genética, Universidad de Valencia, Burjassot, Spain; 2 Instituto de Biomedicina de Valencia, Consejo Superior de Investigaciones Científicas (IBV-CSIC), Valencia, Spain

## Abstract

**Background:**

Classification procedures are widely used in phylogenetic inference, the analysis of expression profiles, the study of biological networks, etc. Many algorithms have been proposed to establish the similarity between two different classifications of the same elements. However, methods to determine significant coincidences between hierarchical and non-hierarchical partitions are still poorly developed, in spite of the fact that the search for such coincidences is implicit in many analyses of massive data.

**Results:**

We describe a novel strategy to compare a hierarchical and a dichotomic non-hierarchical classification of elements, in order to find clusters in a hierarchical tree in which elements of a given "flat" partition are overrepresented. The key improvement of our strategy respect to previous methods is using permutation analyses of ranked clusters to determine whether regions of the dendrograms present a significant enrichment. We show that this method is more sensitive than previously developed strategies and how it can be applied to several real cases, including microarray and interactome data. Particularly, we use it to compare a hierarchical representation of the yeast mitochondrial interactome and a catalogue of known mitochondrial protein complexes, demonstrating a high level of congruence between those two classifications. We also discuss extensions of this method to other cases which are conceptually related.

**Conclusion:**

Our method is highly sensitive and outperforms previously described strategies. A PERL script that implements it is available at .

## Background

**Figure 1:**
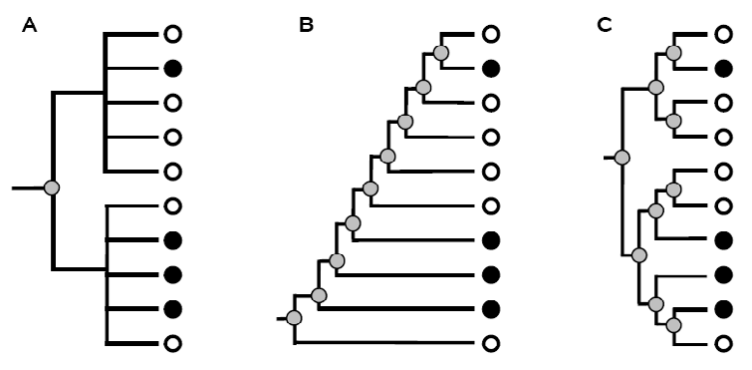
**Comparing a hierarchical and a flat partition of elements in three different contexts**. In these dendrograms, the grey dots indicate the inner nodes that generate clusters to be tested against a dichotomic non-hierarchical classification, which establishes that elements belong (white dots) or not (black dots) to a particular class. In Figure 1A, it is shown the particular case in which two dichotomic flat partitions are compared, but one of them is represented, on the left, as a hierarchical structure. In Figure 1B, a second particular case: provided that the elements in the tree are ordered according to a score, this is equivalent to determining whether the top scorers in an ordered list are enriched for a particular feature. Figure 1C shows a more complex topology, illustrating the general case of comparing a hierarchical and a non-hierarchical classification.

The development of novel methods of data classification – including in this general term both supervised (classification *sensu stricto*) and unsupervised (clustering) methods – is being stimulated by the massive generation of genomic and proteomic data. Classification algorithms can be divided into two categories, hierarchical and non-hierarchical, also known as partitioning [1]. A problem of very general interest is how to compare a hierarchical classification with a dichotomic "flat" classification, i.e. a partition that divides the elements into two non-overlapping groups. Actually, many apparently unrelated cases exist that are particular examples of this general situation (Figure 1). A typical case, and a basic problem in microarray data analysis, is when genes are divided, according to a threshold value, into either differentially expressed or non-changed in an experimental sample respect to a control and then the differentially expressed genes are tested for enrichment in a particular cellular function, for example as defined by those genes being annotated or not with a Gene Ontology (GO) term (e.g. [2-6]). This situation implies comparing two flat classifications. On one hand, genes are divided into genes differentially expressed and genes with background level of expression. On the other hand, genes are partitioned into those annotated and those not annotated with the GO term. However, this is equivalent to compare a flat classification with a hierarchical one, provided that only the deepest dichotomy of the latter (which in this example corresponds to the two classes "differentially expressed" and "not changed") is tested (Figure 1A). A second typical example is how to establish whether the top items of a list, ordered according to a particular parameter, are more often than expected characterized by having a second, independent feature. Taking again an example from the microarray analysis field, this would correspond to a case in which genes are ordered according to a value that measures their level of overexpression (or underexpression) in an experimental sample respect to a control and then it is decided to check whether there is an enrichment of a particular function (e.g. again defined according to the GO classification) among the top scorers (see [7-11]). This situation can also be envisaged as the comparison of a hierarchical and a flat classification, provided that the hierarchical classification (that in this case we want to make equivalent to an ordered list of genes) has the particular asymmetrical structure shown in Figure 1B. Significantly, there is a very extensive literature that explores the two cases summarized in Figures 1A and 1B (reviewed in [12-14]), but it is always in isolation, without considering whether the methods developed for these particular cases could be applied to the general case of hierarchical classifications with more complex topologies (Figure 1C).

To our knowledge, in the whole bioinformatics literature there have been only four recent studies in which it has been attempted to establish general methods to compare hierarchical and non-hierarchical classifications, all of them in the context of microarray data analysis. In two of these studies [15,16], the method is similar, and very much related to those used for the two simpler cases discussed above and exemplified in Figures 1A and 1B. Starting with a hierarchical classification of expression data, which may be obtained with any conventional method, such as UPGMA, the degree of enrichment for a particular class (i.e. a group derived from a flat classification such as whether a gene product is annotated or not with a particular GO term) for each cluster is estimated by calculating the probability *p *of finding such enrichment by chance, using either a cumulative hypergeometric distribution, the equivalent Fischer's exact test or a cumulative binomial distribution. Then, the most significant cluster, the one with smallest *p *value, is determined and all clusters that contain any element in common with it ("parent clusters" and "child clusters", according to whether they contain or are contained in the most significant cluster) are eliminated. The process is repeated until all non-overlapping clusters with small *p *values are determined. Finally, Bonferroni's correction is used to take into account the effect of multiple tests either considering the number of classes tested [15] or the number of clusters tested [16]. A third study followed the same strategy, but only up to the calculation of the *p *values, without further refinement of the results [17]. Finally, a fourth study [18] followed a totally different strategy, based on establishing a heuristic search for minimization of edge crossings in the bigraph generated by the two classifications.

We became interested in this topic after generating a strategy, implemented in the program UVCLUSTER [19] that allows the efficient conversion of complex graphs into dendrograms. We recently used this strategy of analysis both on graphs derived from protein-protein interaction data [19] and on those based on protein domain data [20]. Although we determined that the results obtained in those two works were biologically meaningful, an obvious question to be solved was to establish a standard procedure to determine whether the hierarchical classification obtained was congruent with other classifications (such as GO, division in protein complexes, etc). In this work, we describe a method that follows on the steps of previous studies [15,16], but improves the characterization of the significant classes by using permutation tests that take into account the topology of the hierarchical classification. The method is applied to several cases and, most especially, to explore a hierarchical representation of the mitochondrial interactome, characterizing the clusters that correspond to known protein complexes.

## Results

### Algorithm

Our goal is to detect the clusters of a hierarchical tree that contain an overrepresentation of elements belonging to a particular class. A class is defined by a dichotomic flat partition of the elements in such a way that each element in the tree either belongs or not to it. The likelihood of finding a particular level of enrichment by chance must be evaluated and, in this evaluation, we want to consider the topology of the tree. As we describe in detail below, evaluation is based on a quantitative comparison of the observed enrichment value with the enrichment values of a set of simulated results, generated by random permutation of class labels while keeping constant the topology of the tree.

Let us call *m *the number of elements in the tree. Then, the total number of clusters in that tree is *m *- 1. For each cluster, we can calculate the probability *p *of finding by chance a particular enrichment for a class. To do so, we calculate a cumulative hypergeometric function:

p=∑c=rmin⁡(n,k)(nc)(m−nk−c)(mk)
 MathType@MTEF@5@5@+=feaafiart1ev1aaatCvAUfKttLearuWrP9MDH5MBPbIqV92AaeXatLxBI9gBaebbnrfifHhDYfgasaacPC6xNi=xI8qiVKYPFjYdHaVhbbf9v8qqaqFr0xc9vqFj0dXdbba91qpepeI8k8fiI+fsY=rqGqVepae9pg0db9vqaiVgFr0xfr=xfr=xc9adbaqaaeGacaGaaiaabeqaaeqabiWaaaGcbaGaemiCaaNaeyypa0ZaaabCaKqbagaadaWcaaqaamaabmaabaqbaeqabiqaaaqaaiabd6gaUbqaaiabdogaJbaaaiaawIcacaGLPaaadaqadaqaauaabeqaceaaaeaacqWGTbqBcqGHsislcqWGUbGBaeaacqWGRbWAcqGHsislcqWGJbWyaaaacaGLOaGaayzkaaaabaWaaeWaaeaafaqabeGabaaabaGaemyBa0gabaGaem4AaSgaaaGaayjkaiaawMcaaaaaaSqaaiabdogaJjabg2da9iabdkhaYbqaaiGbc2gaTjabcMgaPjabc6gaUjabcIcaOiabd6gaUjabcYcaSiabdUgaRjabcMcaPaqdcqGHris5aaaa@5032@

In this formula, *m *is, as we just said, the total number of elements in the tree; *n *is the number of elements among those *m *that are included in a class, according to a defined flat partition; *r *is the number of common items between the class and the cluster analyzed; finally, *k *is the cluster size.

**Figure 2:**
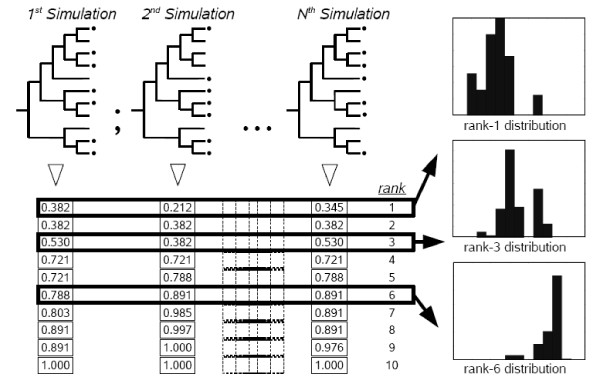
**Method to obtain the expected distributions for *p***. Simulations are performed by randomly permuting class labels while keeping the tree topology constant and cumulative hypergeometric values are ranked to obtain rank-specific random distributions of the *p *value. Although it is not indicated in the figure, results for each rank are obtained from independent sets of simulations.

Our strategy starts by defining all the clusters in a tree and calculating their *p *values. Then, the clusters are ordered according to those values, from minimum to maximum, and the cluster with the minimum value is selected. Now, to establish a null probability distribution of *p *for this first cluster, the program performs a random permutation of class labels. Most significantly, the simulations to generate this distribution take into consideration the topology of the original tree, which is kept constant throughout the permutation process (Figure 2). The probability of finding the observed enrichment is determined by establishing the number of times that the minimum value of *p *in the set of simulations is lower or equal than the observed one. If that corrected probability (*p*') is lower than a particular threshold (e.g. *p*' < 0.01), the cluster is labelled as significant. As a rule of thumb we recommend, when the number of elements of the tree is smaller than 1000, about 1000 permutations to establish a significance value of *p*' lower than 0.05, and no less than 10000 for a value of *p*' < 0.01.

The process described for the first cluster is then repeated for all the rest, going from that with the second lowest *p *value to the one with the maximum *p *value. That is, in each case, the *p *value of the cluster that is analyzed is compared with the *p *values of those clusters found in the same relative position in a new set of simulations (Figure 2) and *p*' values are determined. A significant point is that, given that each ranked value is compared with the values of the same rank of sets of independent simulations, we avoid the need of a further correction for multiple comparisons (the same idea was applied in a different context in [21]).

Once all the results are obtained, it is necessary to filter the results to avoid multiple significant correlated clusters. The rule used is that a significant cluster eliminates all the clusters in the tree that contain any element in common with it ("parent" and "child" clusters) with less significant *p *values. Finally, all significant clusters in which the number of elements belonging to the class that is being considered is lower than 2 are also discarded.

This method has been implemented in a PERL script which is freely available, together with instructions for using it, in our web page [22]. This script may be used for analyses as those shown in the next section, i.e. with up to 500–1000 elements. As an example, the mitochondrial interactome analyses shown in the last section of this article, focused on detecting classes in a tree of 308 elements, required on a standard personal computer from 29 to 188 minutes (average 112 minutes) with 10000 permutations. This range of times is related to how soon significant clusters are found in the particular case examined. We are currently developing a C program called TreeTracker (Arnau, Marco and Marín, in preparation) to be used in cases in which more than 1000 units must be analyzed. Although the program is still not fully optimized, its current version already allows to study large trees in relatively short times. We have performed analyses with a tree of 4860 elements generated from microarray-derived transcriptional data for *Saccharomyces cerevisiae *genes. Its exploration, again with 10000 permutations, required an average of 199 minutes (range 44 – 456 minutes) on a standard PC. Permutations can be easily divided among multiple computers or processors and therefore the analyses can be speed up using more sophisticated hardware.

### Examples of application of this novel strategy

To check for the ability of our new strategy to detect significant similarities between a hierarchical and a flat classification, we have analyzed several real cases. We also tested how our method compares to the already published procedures to determine whether it significantly improves on them.

#### Comparison of a hierarchical classification based on coexpression data and a flat classification based on GO

**Figure 3:**
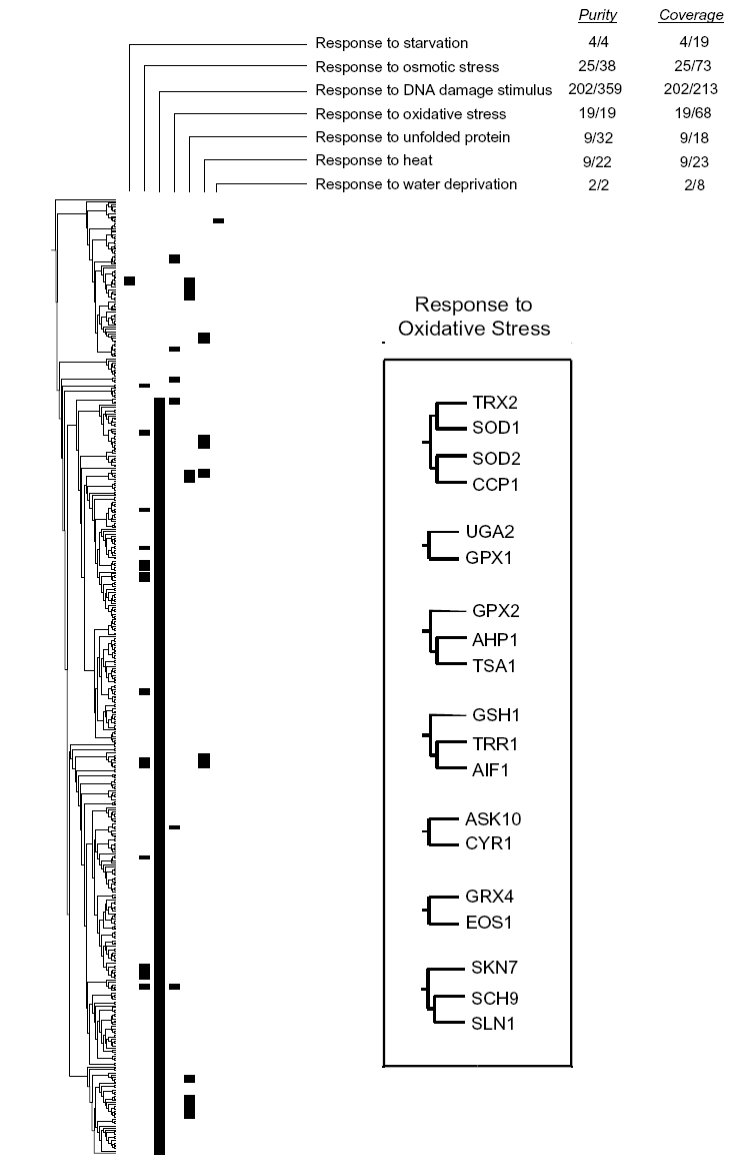
**Summary of the significant clusters obtained for several "Response to stress" GO classes**. Significant clusters are indicated with black rectangles. Purity and coverage for the seven GO classes that generated positive results are indicated. As an example, the box shows the 7 positive clusters for the "Response to oxidative stress" GO class, which include 19 genes, all of them belonging to that class. The discovery of all these small significant clusters in a tree that contains 454 genes demonstrates the sensitivity of the method.

In this first example, we compared a hierarchical classification based on gene coexpression data obtained using microarrays and flat classifications based on establishing whether those same genes belong or not to particular GO classes. We chose to analyze a well-known dataset. Gasch et al. [23] obtained data for the transcriptional response of *Saccharomyces cerevisiae *cells when they were exposed to diverse environmental changes. From the 142 microarrays of this dataset, we extracted information for 454 *S. cerevisiae *genes annotated as belonging to the general GO class "Response to stress". Those genes were hierarchically clustered (see Methods) and then we explored using our strategy whether 10 different GO classes, all them included in the general class "Response to stress" (see list also in the Methods section), were overrepresented in clusters of the tree. Results are summarized in Figure 3. A total of 28 significant clusters were detected for 7 of those classes (detailed in Figure 3), while no clusters were detected for the other three ("Response to cold", "Response to hydrostatic pressure" and "Regulation of transcription in response to stress", which are three small GO classes containing respectively 7, 2 and 3 elements). In this case, the *coverage*, defined as the percentage of genes in the whole dataset that are detected in significant clusters was 85.4 %, and the *purity*, defined as the percentage of proteins in the significant clusters that belonged to the corresponding GO classes, was in average 70.2 %, ranging from 28.1 to 100 %. Very significantly, in this analysis we detected only four significant clusters following the procedures of Toronen [15]. These four clusters were also detected by our method. Moreover, three of those four clusters had size < 5. Toronen did not considered clusters of size < 5 in his original report, but if only clusters of size ≥ 5 are considered, a single cluster is detected by his method. Following Buehler *et al*. method [16], we only detected two clusters, also both found by our strategy. In summary, the previous methods detected at most 4 clusters while ours detected the same clusters plus 24 additional ones. We conclude that our strategy is much more sensitive. The difference is clearly due to the fact that Bonferroni's correction (used in those two previous works) is too strict, an effect that becomes more and more noticeable when the number of tested classes increases. It is obvious that this qualitative advantage in sensitivity clearly compensates for the fact that our method is more computer intensive.

#### Comparison of coexpression data versus protein complexes

It is widely accepted that, at least in yeasts, proteins appearing together in a protein complex are likely to be encoded by genes with a degree of coexpression higher than expected by chance [24,25]. Thus, as a second example of our strategy, we decided to search in a hierarchical tree based on coexpression data for clusters in which genes encoding proteins of particular complexes were overrepresented. To do so, 34 protein complexes, containing a total of 207 proteins, were arbitrarily selected from SGD (see Methods; for a complete list, see Additional File 1 Table 1). Then, we grouped, using hierarchical clustering based on coexpression data (see again Methods for the details), the 207 corresponding genes. Applying our novel strategy to compare both datasets, we detected significant associations for 15 out of the 34 protein complexes. The coverage was here 24.6 % (51/207; with the coverage of particular complexes ranging from 0 to 75 %; see Additional File 1 Table 1) and the purity was in average 37.0 % (again see Additional File 1 Table 1). These results show that, in this particular dataset, the classification based on coexpression is only partially congruent with the classification based on protein complexes. This relatively low level of congruence and the fact that the complexes were in general of small size (average 6.1 proteins/complex) makes difficult the characterization of significant clusters. However, our results (15/34 = 44% of complexes detected as significant) are again qualitatively better than those provided by the other related strategies, because in this case we failed to detect a single significant cluster following the procedures of Toronen [15] or Buehler *et al*. [16], even if cluster sizes < 5 are considered.

#### Comparison of two unsupervised clustering processes

Another type of situation in which it is relevant to compare a hierarchical and a non-hierarchical classification is found when two different methods of clustering, one of them hierarchical and the second not, are used with the same data. For example, it is often significant to establish whether a hierarchical clustering result is compatible with a k-means-based flat partition. To see how our strategy performed in this case, we randomly select microarray data for a set of 200 yeast genes (see Methods) and obtained a hierarchical UPGMA tree [26], and two k-means classifications [27], in which the results were fitted into 10 or 20 clusters, respectively. In this example, we obtained significant hierarchical clusters for all the either 10 or 20 classes defined by the k-means partitions. However, when 10 classes were used, only 2 of them had corresponding single significant clusters, while the other 8 produced multiple separated significant clusters. On the other hand, when 20 classes were established with k-means, we found that 12 of them corresponded to single significant clusters. The weakest point in the k-means strategy of partition is the need of an *a priori *definition of the number of classes and our strategy can be useful to establish the optimal value for that number. In our example, it was clear than the division into 20 classes was more similar to the hierarchical classification of the same elements that a division into 10 classes. This type of comparison may be thus used both as to roughly establish the best number of clusters in which to divide a group of elements by k-means analyses and also to determine which ones of those clusters are supported by both hierarchical and k-means classifications.

### The structure of the yeast mitochondrial interactome

**Figure 4:**
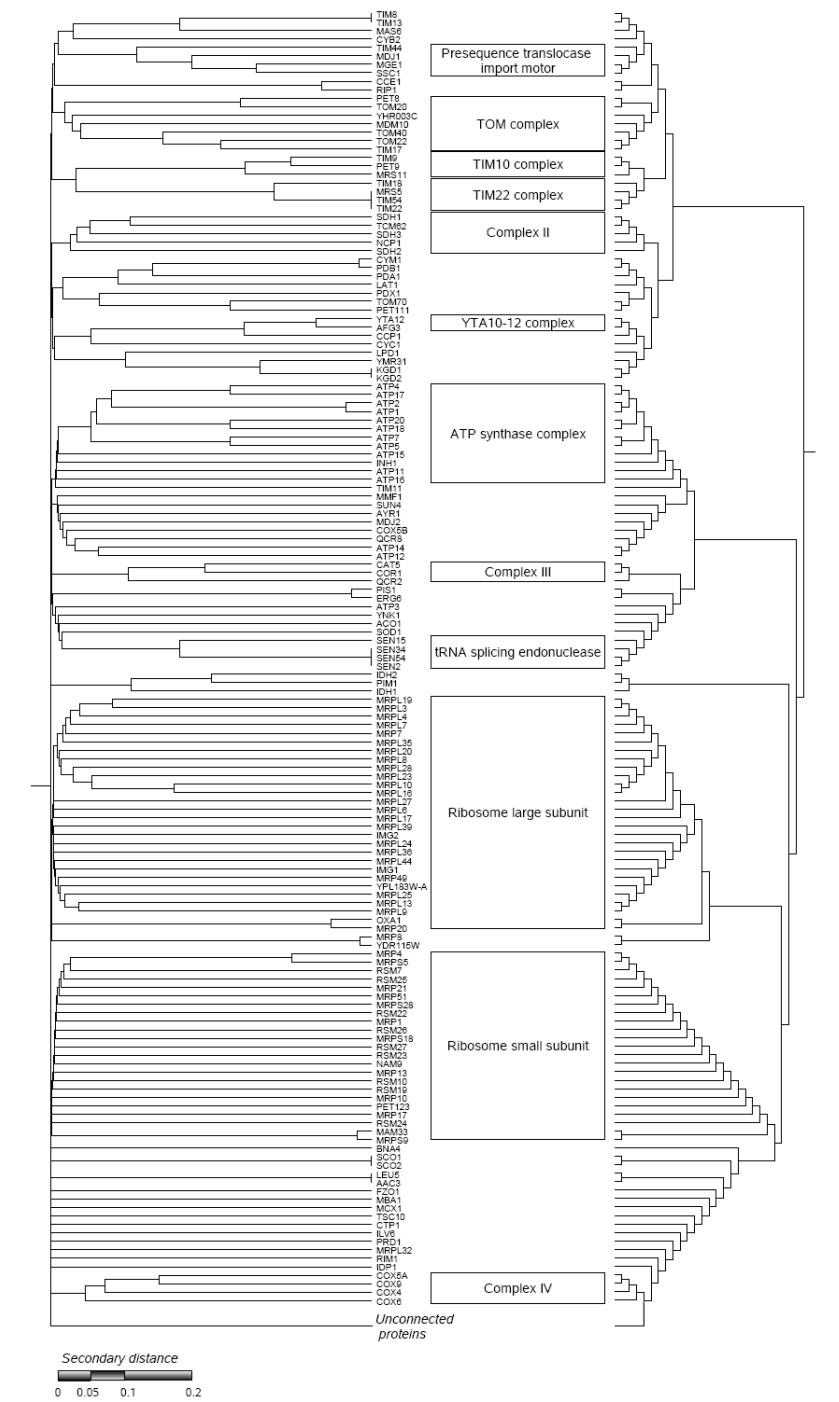
**Hierarchical representation of the yeast mitochondrial interactome and details of the clusters overrepresented for proteins of particular complexes**. The trees were obtained with UVCLUSTER. The tree on the left has branches that are proportional to the distances while the one on the right serves to show more clearly the underlying topology.

We finally examined in detail a more difficult case, to see if it was possible to successfully recover the structure of part of a complex interactome by using UVCLUSTER analyses, which builds a hierarchical structure starting from a graph [19]. We thus compared a UVCLUSTER-based hierarchical dendrogram derived from general protein-protein interaction data for mitochondrial yeast proteins with a flat partition based on known mitochondrial protein complexes. A total of 308 proteins were considered (see Methods). Out of 16 protein complexes tested, 12 of them (75%) were detected as significant clusters in the hierarchical tree (Figure 4). A thirteenth (the mitochondrial inner membrane peptidase complex, containing proteins IMP1 and IMP2) was located at the bottom of the tree together with unconnected proteins and therefore it was considered not significant. This situation occurs because, as often happens in this type of biological graphs [28], most proteins are connected, forming a large core. Unconnected proteins are those not directly linked to any protein of that main core, and therefore they appear as an artifactual cluster in the hierarchical trees.

In this case, the coverage was 75.5% and the purity of the clusters was 88.3%. Therefore, the correlation between the hierarchical and the flat partition was very high, demonstrating that significant portions of the yeast interactome can be meaningfully characterized using unsupervised, fully automated UVCLUSTER hierarchical clustering.

The comparison of the hierarchical, global, structure of the protein-protein interaction graph with the known protein complexes provides two different levels of novel knowledge. First, we can visualize the relationships between elements inside a protein complex. Second, we can study the global hierarchical relationships among protein complexes. Several well-known relationships among complexes are observed in Figure 4, being the most obvious the close proximity between the subunits of the large and small ribosomal subunits. Similarly, we can see the close relationships among different complexes involved in protein translocation from the cytosol to the mitochondria, such as the TOM complex, the Tim10 and Tim22 complexes and the presequence translocase-associated import motor (see [29]). The analysis of the congruence of the two types of data may also provide novel information about proteins of unknown function. For instance, the cluster that contains known members of the TOM complex (Tom20, Tom22 and Tom40; [29]) contains four additional proteins: Pet8, Mdm10, Tim17 and the unknown protein YHR003c. Our analysis suggests that a functional relationship among these proteins and the TOM complex is likely and in fact data exist confirming this relationship for two of the proteins: Mdm10 is involved in the assembly of the TOM complex [30] and Tim17 has been recently described as a mediator between the TOM complex and the presequence translocase import motor [31] (both closely related in our tree, see Figure 4). Pet8 is known to be involved in the transport of S-adenosylmethionine (SAM) into the mitochondria, although the molecular mechanism is still unknown [32]. Our results suggest a possible involvement of the TOM machinery in SAM transport, with Pet8 linking both processes. Finally, YHR003c has been located to the mitochondrial outer membrane in a massive screen [33], which is congruent with an interaction with the TOM complex. Interestingly, YHR003c has a high similarity with the ThiF family of proteins, which in eukaryotes comprise a large family of ubiquitin-activating enzymes [34] suggesting a possible control of these aspects of the mitochondrial protein transport by the ubiquitin-proteasome system.

Of course, congruence is not perfect. For example, other known members of the TOM complex, such as Tom6, Tom7 and Tom70 are outside the corresponding significant cluster. However, inspection of our datasets demonstrated that this is due to the fact that protein-protein interaction data for these proteins and the rest of the TOM complex is lacking in the DIP database, and therefore we can attribute this problem to incompleteness of the data used to generate the hierarchical tree. In any case, all these results demonstrate that our strategy of analysis is useful to detect relevant correlations between a hierarchical and a non-hierarchical partition of the same data and that UVCLUSTER can be used to extract significant portions of a complex graph in order to determine its hierarchical structure, confirming our previous findings [19,20].

## Discussion

In this paper, we describe a new strategy that allows establishing the clusters of a hierarchical tree that are congruent with a non-hierarchical classification of elements. The main novelty of our strategy is the use of permutation analyses, which generate a distribution of ranked probability values, to check for significantly enriched clusters. The method outlined here has the main advantage of being more sensitive than similar, previously published methods [15,16] due to taking into consideration the topology of the tree to evaluate the likelihood of obtaining by chance each level of overrepresentation. The advantage of our method is especially evident in cases in which many classes are analyzed, due to the fact that Bonferroni's correction, used by those authors, is then too conservative.

There are many papers already published using permutation methods to establish the significance levels of enrichment for the particular cases shown in Figures 1A (e.g. [3,4] and many others) and Figure 1B (e.g. [10,11]). It is relevant to point out here that our method reduces to those other methods provided that the hierarchy has the particular structure shown in those figures. This is obvious for the case depicted in Figure 1A, in which only one test is performed. However, it is also true for the more complex case in Figure 1B. It can be analyzed with our strategy and will provide exactly the same results that those already established by other methods, in spite of the fact that here we use a ranking for the significant clusters. The reason is that, if, and only if, the hierarchical structure is as shown in Figure 1B, the maximally significant cluster (i.e. the one with the smallest *p*) eliminates all the other possible clusters, because all the rest are "parents" or "children" of it. Our study may thus be considered the logical conclusion of a line of research that has been developed in the last years without actually being studied in the right, general context. It is the first one in which it is shown that those two are just particular cases of the general situation in which a hierarchical and a dichotomic flat partition are compared, and a general solution for such comparison is offered.

As we have indicated in the introduction of this work, all published methods that we know of in which hierarchical and non-hierarchical classifications are compared are restricted to the field of microarray data analysis. We have shown above an example of its use in a different context, namely protein-protein interaction data, and of course this method may be used in many other different contexts to undertake biological or non-biological problems. In particular, the combination of UVCLUSTER, which establishes the hierarchical structure that more faithfully reflects a graph, and this strategy, which may be used for establishing the meaning of that hierarchical structure, allows for the analyses of complex graphs in ways that had not been hitherto possible.

## Conclusion

Here we present a new strategy for the comparison of a hierarchical and a non-hierarchical classification of elements. This strategy can be applied to very different situations, among them the particular cases of comparing flat classifications (e.g. functional information) with two lists of genes (e.g. Experimental vs. Control), or with an ordered list of genes. The main improvement is that our strategy considers the topology of the tree during the calculation of significance levels. The use of permutation analysis and the rank-based comparisons of *p *values allows the method to be highly sensitive.

## Methods

### Microarray data

Expression data for the comparison of coexpression analysis vs. GO were extracted from [23]. The ten GO classes examined were "Response to heat", "Response to cold", "Response to DNA damage stimulus", "Response to oxidative stress", "Response to water deprivation", "Response to osmotic stress", "Response to unfolded protein", "Response to starvation", "Response to hydrostatic pressure" and "Regulation of transcription in response to stress". All them are included in the general class "Response to stress" that was used as criterion to select the 454 genes that were clustered. GO information for this and the rest of analyses shown in this work was obtained from SGD [35]. Hierarchical clustering of expression data was performed using euclidean distances and the UPGMA method, with MeV 4.0 [36] using its default parameters [37]. For the comparison of coexpression analysis and protein complexes the data were extracted from [38], a dataset that includes transcriptional information for experiments involving 300 different mutations or treatments in *Saccharomyces cerevisiae*. Expression data used in the comparison of k-means vs. UPGMA classifications were all extracted from the webpage of Michael Eisen's laboratory [39]. Both hierarchical and k-means clustering of microarray data were performed again with MeV 4.0 with default parameters.

### Protein complex information

Protein complex information were also extracted from SGD. In the comparison involving coexpression data, we randomly selected 34 protein complexes that included a total of 207 proteins. In the analysis of the yeast mitochondrial interactome, we considered a total of 16 protein complexes annotated in SGD as mitochondrial.

### Generation of the hierarchical dendrogram for the yeast mitochondrial interactome

A protein interaction network of the *Saccharomyces cerevisiae *mitochondrial interactome was built by extracting the 438 proteins annotated as mitochondrial in SGD and obtaining all the protein-protein interactions among them reported in the DIP database [40]. Protein interaction information was available for 308 proteins. The resulting interaction network was analyzed with UVCLUSTER [19] (parameter *AC *= 100; 10000 iterations) to obtain a hierarchical representation of the graph.

### Applications of our strategy

For all analyses described in that work, we performed 10000 simulations in order to establish the expected distributions and the critical values for *p*' < 0.01.

## Authors' contributions

The strategy was developed by both authors. A.M. generated the analyses presented here and wrote the Perl script that implements this strategy. He also contributed to the manuscript, which was written by I.M. Both authors read and approved the final manuscript.

## Supplementary Material

Additional file 1 Additional File 1 Table 1. Details of the protein complexes used to compare to the dendrogram of coexpression of their genes. Click here for file
