# Supplementary material for: A general strategy to determine the congruence between a hierarchical and a non-hierarchical classification
Source: BMC Bioinformatics. 2007 Nov 15;8:442. doi: 10.1186/1471-2105-8-442 (PMC2213689; doi:10.1186/1471-2105-8-442)
Supplement: Additional file 1 — Table 1. Details of the protein complexes used to compare to the dendrogram of coexpression of their genes. [file 1471-2105-8-442-S1.doc]

**Supplementary Table 1.** Protein complexes used to compare to the dendrogram of coexpression of their genes. See main text for details about *purity* and *coverage* statistics. Asterisks indicate that no significant cluster was detected using our strategy.

| Protein Complex | complex size | purity (%) | Coverage per complex (%) |
| --- | --- | --- | --- |
| 1,3-beta-glucan synthase complex | 2 | * | * |
| Ada2/Gcn5/Ada3 transcription activator complex | 5 | * | * |
| AMP-activated protein kinase complex | 5 | * | * |
| AP-1 adaptor complex | 6 | * | * |
| AP-2 adaptor complex | 4 | * | * |
| AP-3 adaptor complex | 4 | 2/7 (28.6) | 2/4 (50.0) |
| Arp2/3 protein complex | 7 | * | * |
| CBF3 complex | 4 | * | * |
| CCAAT-binding factor complex | 4 | 2/3 (66.7) | 2/4 (50.0) |
| CCR4-NOT complex | 12 | 5/6 (83.3) | 5/12 (41.7) |
| CCR4-NOT core complex | 7 | 4/6 (66.7) | 4/7 (57.1) |
| Cdc73/Paf1 complex | 7 | 2/8 (25.0) | 2/7 (28.6) |
| COMPASS complex | 8 | * | * |
| Cul3-RING ubiquitin ligase complex | 4 | 3/29 (10.3) | 3/4 (25.0) |
| DASH complex | 10 | 4/14 (28.6) | 4/10 (40.0) |
| DNA-directed RNA polymerase I complex | 14 | * | * |
| DNA-directed RNA polymerase II, core complex | 12 | 7/62 (11.3) | 7/12 (58.3) |
| EKC/KEOPS protein complex | 5 | * | * |
| Golgi transport complex | 8 | 2/2 (100) | 2/8 (25.0) |
| GPI-anchor transamidase complex | 5 | * | * |
| HOPS complex | 6 | * | * |
| INO80 complex | 11 | 6/25 (24.0) | 6/11 (54.5) |
| ISW1 complex | 4 | * | * |
| MCM complex | 6 | * | * |
| Mre11 complex | 3 | 2/19 (10.5) | 2/3 (66.7) |
| Ndc80 complex | 4 | * | * |
| RNA polymerase I upstream activating factor complex | 5 | * | * |
| Rpd3S complex | 5 | * | * |
| RSC complex | 17 | 5/18 (27.8) | 5/17 (29.4) |
| Sbr-mediator complex | 7 | * | * |
| Sec62/Sec63 complex | 4 | 3/39 (7.7) | 3/4 (75.0) |
| Smc5-Smc6 complex | 8 | 2/8 (25.0) | 2/8 (25.0) |
| THO complex | 4 | 2/5 (40.0) | 2/4 (50.0) |
| TORC1 complex | 5 | * | * |

Average purity for significant clusters: 37.0 ± 7.4 %

Coverage for the whole dataset: 51/207 = 24.6 %
